# Absolute flatness measurements of silicon mirrors by a three-intersection method by near-infrared interferometry

**DOI:** 10.1186/1556-276X-8-275

**Published:** 2013-06-07

**Authors:** Junichi Uchikoshi, Yoshinori Hayashi, Noritaka Ajari, Kentaro Kawai, Kenta Arima, Mizuho Morita

**Affiliations:** 1 Department of Precision Science and Technology, Graduate School of Engineering, Osaka University, 2-1 Yamada-oka, Suita, Osaka 565-0871, Japan

**Keywords:** Absolute flatness, Silicon, Mirror, Interferometer, Near infrared, Phase shift, Three-flat method, Three-intersection method

## Abstract

Absolute flatness of three silicon plane mirrors have been measured by a three-intersection method based on the three-flat method using a near-infrared interferometer. The interferometer was constructed using a near-infrared laser diode with a 1,310-nm wavelength light where the silicon plane mirror is transparent. The height differences at the coordinate values between the absolute line profiles by the three-intersection method have been evaluated. The height differences of the three flats were 4.5 nm or less. The three-intersection method using the near-infrared interferometer was useful for measuring the absolute flatness of the silicon plane mirrors.

## Background

High-precision measurements of surface flatnesses are important in the development of optical devices. In flatness testing, interferometry with a standard flat is used for high-precision measurements. In a measurement with a standard flat, the measurement accuracy is mainly determined using the figure of the standard flat. The three-flat method by interferometry is commonly used to measure the flatness of standard flat surfaces for high-precision interferometers. This method allows others to measure the absolute line profile, and its importance is widely accepted [1-4]. The absolute testing of optical flats has been discussed by a rotation-shift method [5].

High-grade flats are required for interferometry with a standard flat because the accuracy is critically dependent on the figure. Recently, flattened silicon surfaces on the nanometer scale have been prepared [6-8]. A silicon flat is expected to be one of the standard flats. The absolute line profile of the silicon mirror cannot be measured by the three-flat method when a visible light is used. To measure the absolute line profile of the silicon mirror by the three-flat method, an interferometer with a light source where the silicon mirror is transparent must be constructed, and only three silicon mirrors are used to measure the absolute line profiles. However, the absolute line profile measurement of the silicon mirror with a near-infrared light has not been carried out using only silicon mirrors. A near-infrared Fizeau interferometer with a 1.55-μm wavelength laser diode has been developed to improve the fringe contrast for large surface roughness. However, a near-infrared interferometer using a shorter wavelength has not been tested [9].

The authors constructed an interferometer using a near-infrared laser diode with a 1,310-nm wavelength light where the silicon plane mirror is transparent. They also measured the absolute line profiles of three silicon plane mirrors for standard flats through the use of the three-flat method by near-infrared interferometry [10]. In this paper, the authors describe a method for measuring the absolute flatness of silicon mirrors by a three-intersection method and determine the absolute shapes using a near-infrared interferometer. To evaluate the precision of the absolute flatness measurements, the authors examine the height differences in the absolute shapes.

## Methods

**Figure 1:**
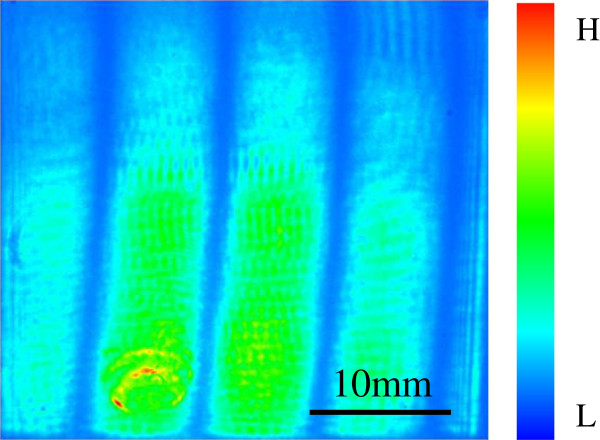
Schematic diagram of the near-infrared interferometer.

**Figure 2:**
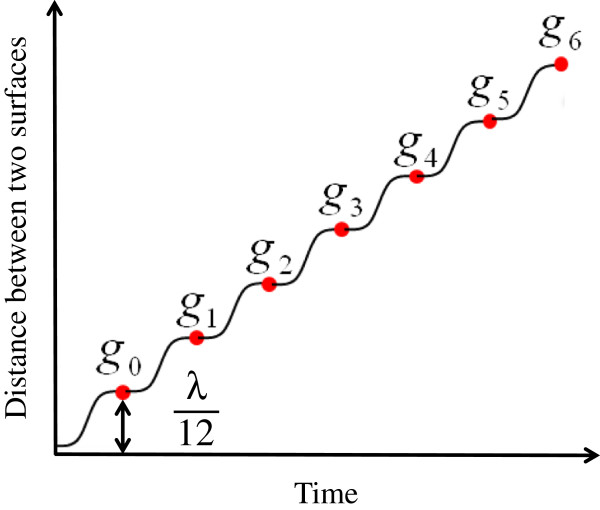
Photograph of the near-infrared interferometer.

Figure 1 shows a schematic diagram of the near-infrared interferometer. The near-infrared interferometer was built based on the Fizeau interferometer. Figure 2 shows a photograph of the near-infrared interferometer. The near-infrared laser diode (FOL13DDRC-A31, Furukawa Electric Co., Ltd., Chiyoda-ku, Tokyo, Japan) with a 1,310-nm peak wavelength light where the silicon plane mirror is transparent, was used as a light source. The typical peak wavelength of the laser light was 1,310 nm. The temperature dependence of the peak emission wavelength was 0.09 nm/°C. The ambient temperature fluctuation during the measurements by the three-flat method was within 0.1°C. The temperature of the laser diode was within 0.1°C. The wavelength fluctuation was estimated to be 0.009 nm from the temperature dependence and fluctuation. The output light from the near-infrared light source was expanded to the necessary size. A parallel light was provided using the collimator and perpendicularly incident on the reference and detected surfaces. The reference and detected surfaces were placed almost parallel, and the distance between them was approximately 24 mm. The light was divided into two waves on the reference surface. One of the waves was reflected on the surface and the other passed through it. The wave passing through the reference surface was reflected on the detected surface. The two reflected waves passing through the imaging lens interfered and formed interferograms. The image of the interferogram was put into a personal computer with a near-infrared charge-coupled device (CCD) camera (C5840, Hamamatsu Photonics K. K., Hamamatsu, Shizuoka, Japan). The CCD camera had a high sensitivity to wavelengths from 400 to 1,650 nm. The signal of the CCD camera output was converted to a 10-bit digital signal using a video analog-to-digital converter. The 32 digital signals were accumulated on a computer with a software (LabVIEW, National Instruments Corporation, Austin, TX, USA) designed to obtain the average. The first 10 digits of the average signal were chosen as the measured value of the interferogram intensity.

**Figure 3:**
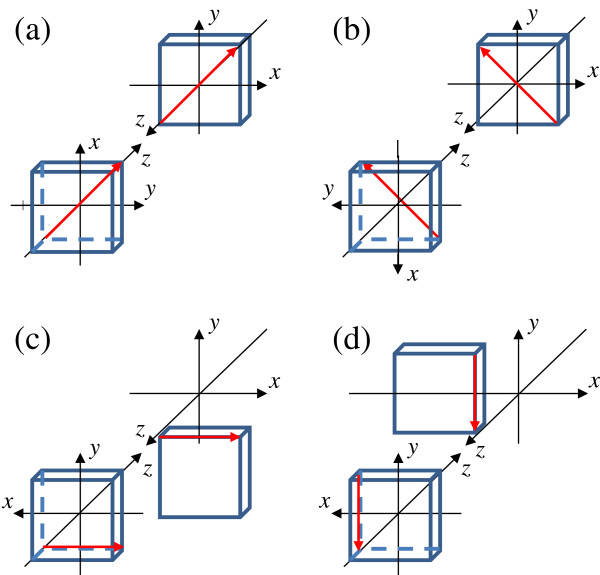
A typical intensity map of an interferogram.

Figure 3 shows a typical intensity map of an interferogram. The distance between the reference and detected surfaces varied by an interval of *λ*/12 to *λ*/2 with a phase shift stage, and interferograms were recorded at equal intervals of the shifted distance using the CCD camera. The phase shift stage which was composed of elastic hinges and a piezoelectric actuator traveled in a straight line. In order to reduce the effects of environmental vibration or temperature drift on the measurement precision, the reference surface was moved stepwise for the phase shift, and the intensities of the interferograms were averaged on a platform at each step. The reference surface was moved by accelerating or decelerating the drive with the phase shift stage under low acceleration just after starting or before stopping to avoid the drift of the reference surface caused by vibration. Environmental vibration was attenuated using an active vibration-isolated table (AVI-350M, Herz Co., Ltd., Yokohama, Kanagawa, Japan). The acceleration of the environmental vibration was approximately 2 mgal. Both the reference and detected surfaces were silicon plane mirror surfaces. The silicon plane mirror was a square plate with polished surfaces on both sides. To prevent interference by the reflected light from the back surface of the reference or the detected surface, a wedge was formed on the back surface of the silicon plane mirrors. The designed width, thickness, wedge angle, and azimuth angle of the wedge were 50.0 mm, 10.0 mm, 0.28°, and 22.5°, respectively. The silicon plane mirrors were polished with a magnetorheological finishing (MRF) [11], and the flatnesses were 30 nm or less. The silicon plane mirror was supported at six points on the sample holder which was fixed on the phase shift stage, and the mirror was supported at three points on the back surface, two points on the undersurface, and one point on the side surface.

**Figure 4:**
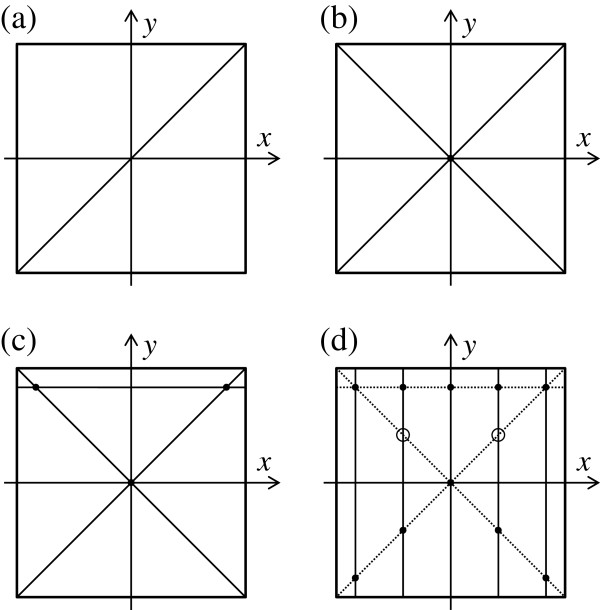
Sampling for the 6 + 1-sample algorithm.

From the interferogram intensities at each pixel site of the CCD camera, the initial phase of each pixel site was calculated by 6 + 1-sample algorithm [12]. Figure 4 shows the sampling for the 6 + 1-sample algorithm by the following equation:

(1) $$\theta =\tan^{-1}\left\{\sqrt{3}\;\left(\frac{g_{1}+g_{2}-g_{4}-g_{5}+\frac{g_{6}-g_{0}}{3}}{-g_{0}-g_{1}+g_{2}+2g_{3}+g_{4}-g_{5}-g_{6}}\right)\right\}.$$

The relative heights of the reference and detected surface were calculated from the initial phases and the wavelength. Three silicon plane mirrors (A, B, and C flats) were combined in pairs with different positional combinations (transmission reference A and detected B, A and C, and B and C) in the interferometer and used for calculation of the absolute line profile of each silicon plane mirror by the three-flat method [2]. The absolute line profile could be measured only along a vertical center line on the reference and detected flats. The B flat in the combination B and C was rotated around the vertical center line compared to the B flat in the combination A and B. The position of the center and the direction of the center line on the detected flat were adjusted to be the same as those on the reference flat within 1 pixel of the CCD camera (which has 640 × 480 pixels). One pixel corresponds to 107 μm on the flat.

**Figure 5:**
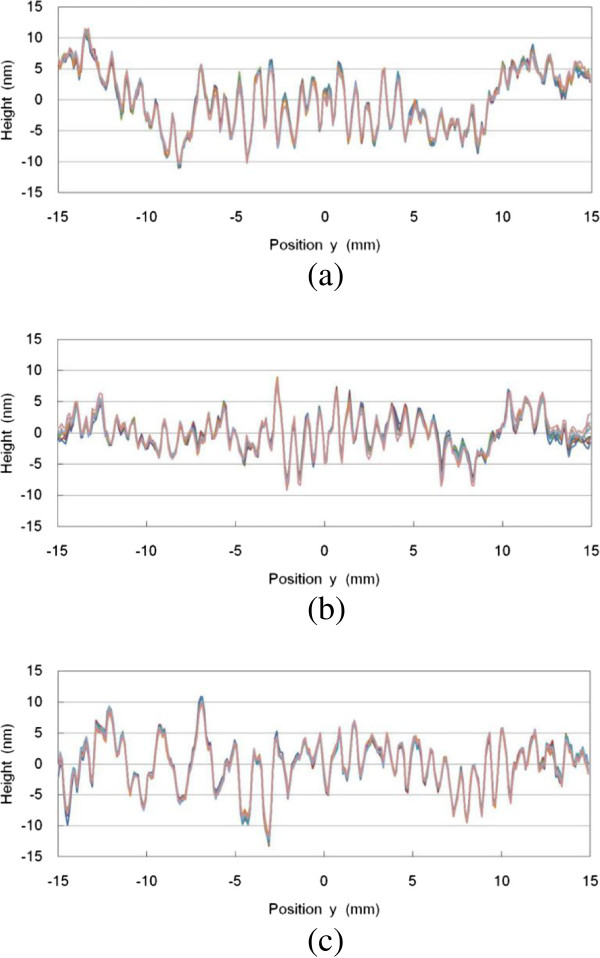
**Arrangement of the reference (lower left) and detected (upper right) flats in the three-intersection method.** For (**a**) rotation axis on diagonal, (**b**) another diagonal, (**c**) line at *y* = 10.0 mm, and (**d**) line at *x* = 10.0 mm.

**Figure 6:**
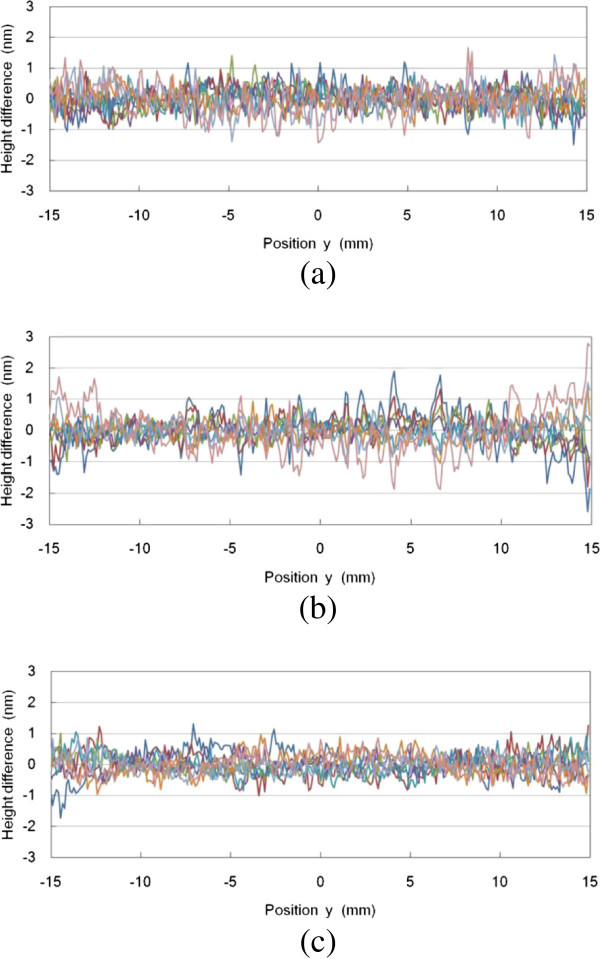
**Test configurations in absolute flatness measurements by the three-intersection method.** For (**a**) rotation axis on diagonal, (**b**) another diagonal, (**c**) line at *y* = 10.0 mm, and (**d**) lines at *x* = -10.0, -5.0, 0.0, 5.0, and 10.0 mm.

Figure 5 shows the arrangement of the reference and detected flats in absolute flatness measurements by the three-intersection method. Both rotating and shifting were used to eliminate an indeterminate term that equated to a twisted surface [13]. In the three-intersection method, the reference flat (the B flat) was rotated around the *z*-axis, or the detected flat (the C flat) was shifted from the combination B and C flats in the three-flat method. By rotating the reference flat 90 or -90° clockwise around the *z*-axis, as shown in Figure 5a,b, the absolute line profile could be measured only along a diagonal or another diagonal line on the reference and detected flats. By shifting the detected flat to *y* = -20.00 mm or *x* = -20.00 mm using the XZ stage (FS-1100PXZ, SIGMA TECH. CO., LTD., Hanno, Saitama, Japan), as shown in Figure 5c,d, the absolute line profile could be measured only along a line at *y* = 10.0 mm or *x* = 10.0 mm. Figure 5 shows the test configurations in absolute flatness measurements by the three-intersection method. An absolute line profile could be measured only along a rotation axis on the reference or the detected flat by the three-flat method. Figure 6a,b,c shows the configuration of the rotation axis on a diagonal, another diagonal and a line at *y* = 10.0 mm, respectively. Heights of the three absolute profiles along the three axes were adjusted to be zero at three intersections indicated by solid circles in Figure 6c. Five absolute line profiles along the rotation axes parallel to the *y*-axis were measured at *x* = -10.0, -5.0, 0.0, 5.0, and 10.0 mm in Figure 6d. The height of each profile was adjusted to be the same as that of the profiles at the two intersections indicated by solid circles for *y* = 10.0 mm, one diagonal or for *y* = 10.0 mm, and another diagonal. Thus, an absolute flatness could be measured by the three-intersection method.

## Results and discussion

**Figure 7:**
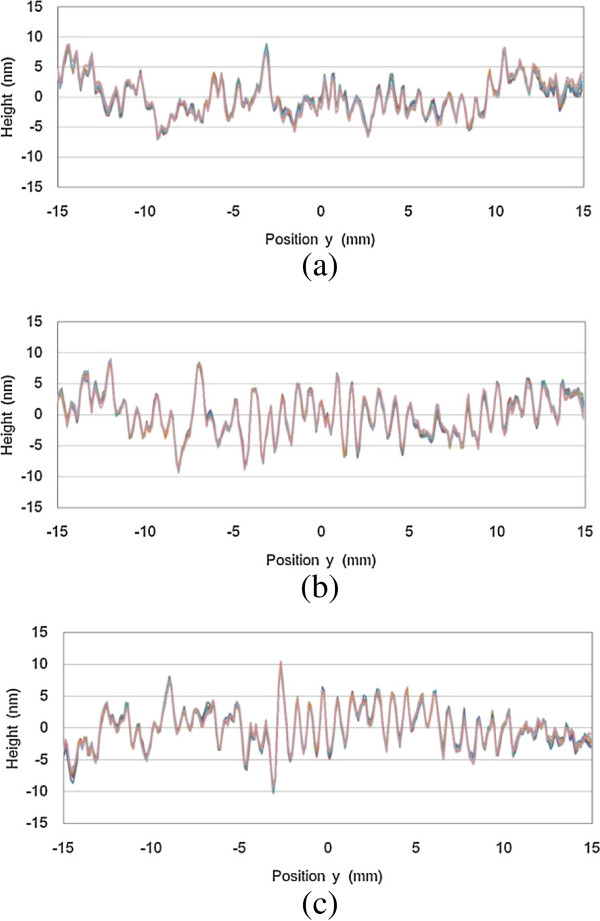
**Relative line profiles along a vertical center line.** For (**a**) A and B, (**b**) A and C, and (**c**) C and B flats.

Figure 7 shows the relative line profiles of the reference and detected surfaces along the vertical center line. The relative line profiles were calculated from a set of interferograms by the 6 + 1-sample algorithm for the one phase-shifting interval of *λ*/6. The *y*-axis is the dimension of the measured length. The length was 30.0 mm. The relative line profiles were calculated for eight measurements. The inclination of the reference and detected surfaces was removed by applying the least-squares method. The peak-to-valley (PV) values of the relative line profiles in Figure 7a,b,c are approximately 22, 18, and 24 nm, respectively.

**Figure 8:**
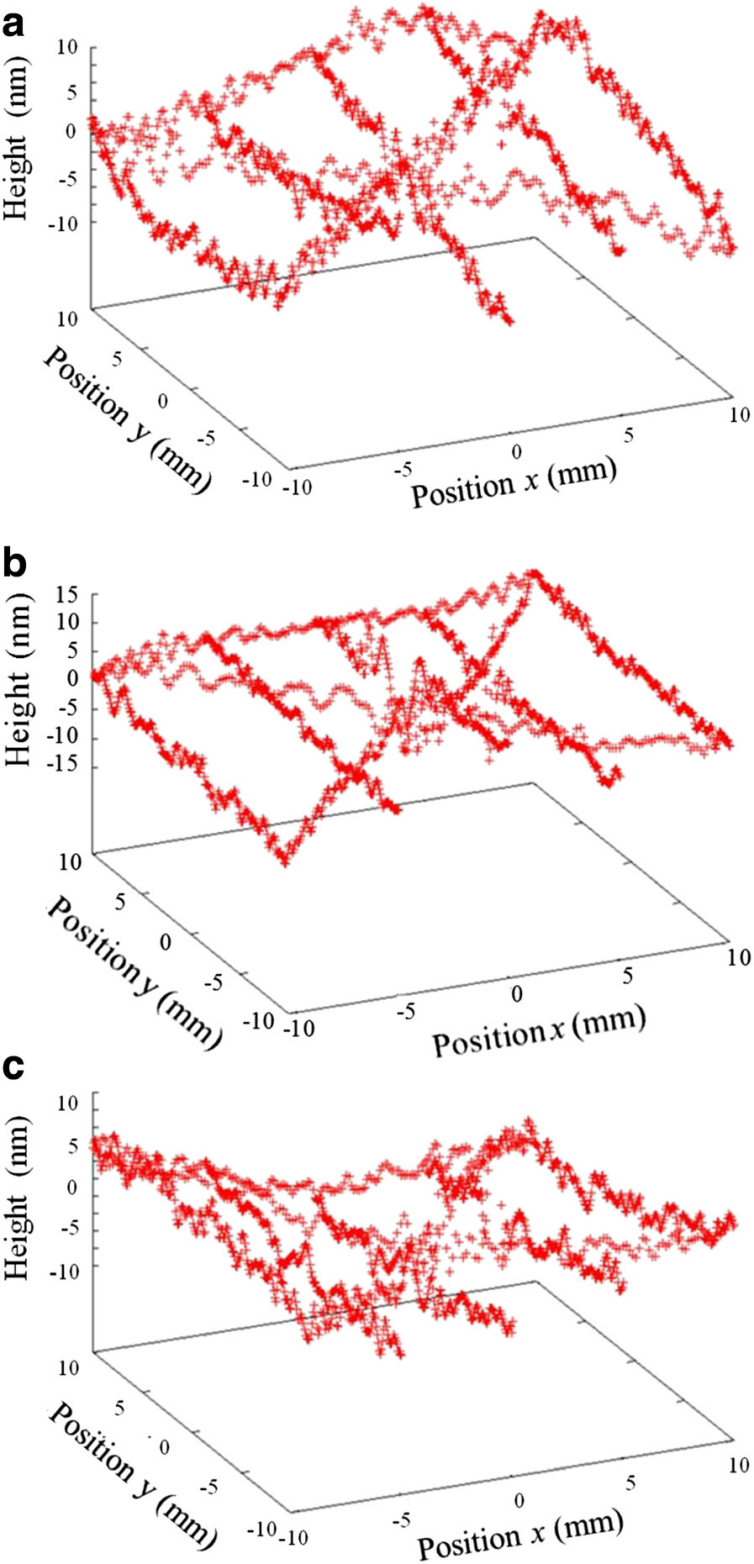
**Height differences between relative line profiles and mean values.** For (**a**) A and B, (**b**) A and C, and (**c**) C and B flats.

Figure 8 shows the height difference between the relative line profiles and the mean value. The height difference was calculated by subtracting one measured profile from the mean value. The PV values of eight height differences were 1.5 nm at minimum and 4.7 nm at maximum. When there was a peak in relative line profiles for one combination of two flats, the corresponding peak could appear in all absolute line profiles of A, B, and C flats in the three-flat method. The root-mean-square (RMS) values of the relative line profiles in Figure 8a,b,c were 0.41, 0.48, and 0.35 nm, respectively. The repeatability of the PV measurements of a commercial interferometer using a visible light of 632.8-nm wavelength was better than *λ*/300. It is necessary to compare the same specification between the near-infrared and visible light interferometers.

**Figure 9:**
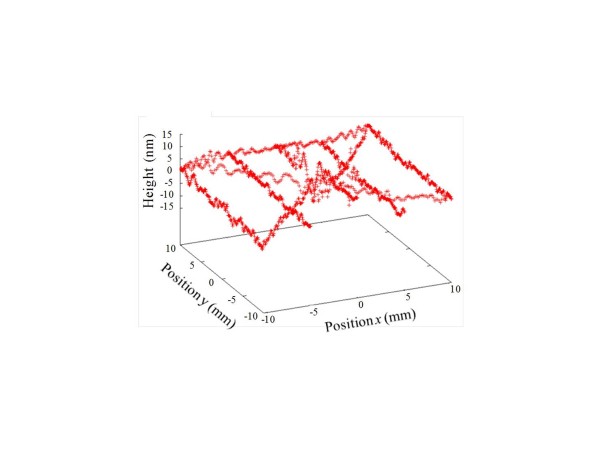
Absolute line profiles of (a) A, (b) B, and (c) C flats for eight measurements.

Figure 9 shows the absolute line profiles of each silicon plane mirror along the vertical center line at *x* = 0.0 mm. The relative line profiles were calculated for eight measurements, and the absolute line profiles were calculated for each measurement. The PV values of the absolute line profiles in Figure 9a,b,c were 15.7, 18.4, and 20.6 nm, respectively. The RMS values of the absolute line profiles in Figure 9a,b,c were 3.0, 3.4, and 3.1 nm, respectively. In Figure 9a,b,c, surface waves are observed. The pitch of the surface waves were approximately 0.75 mm. This suggests that the pitch reflects the feed of the MRF polishing.

**Figure 10:**
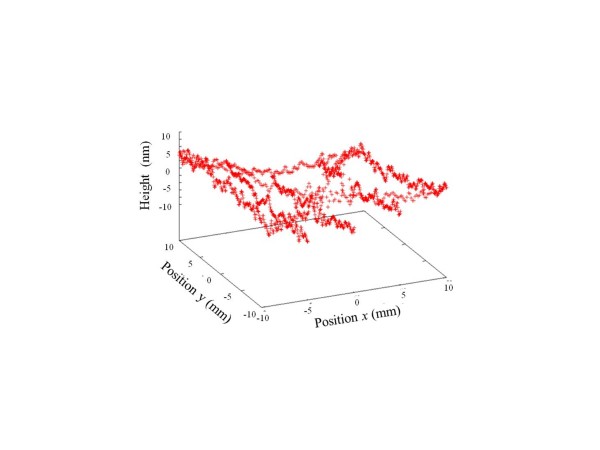
Absolute shapes of (a) A, (b) B, and (c) C flats by the three-intersection method.

Figure 10 shows the absolute shapes of flats by the three-intersection method. Height differences at the *x*-*y* coordinate values (-5, 5) and (5, 5) indicated by open circles in Figure 6d are shown in Table 1. The height differences of the three flats are 4.5 nm or less. The height difference was due to height differences between the relative line profiles and the mean value. This result suggests that the absolute flatness of surfaces can be measured by the three-intersection method by near-infrared interferometry.

**Table 1:** Height differences at coordinate values

***x*-*y *coordinate value**	**Height difference (nm)**		
	A flat	B flat	C flat
(-5, 5)	4.5	4.0	3.4
(5, 5)	1.5	0.4	1.2

## Conclusions

The authors measured the absolute flatness of three silicon plane mirrors with the three-intersection method using the near-infrared interferometer. The height differences at the *x*-*y* coordinate values have been examined to evaluate the precision of the absolute flatness measurement. The height differences of the three flats were 4.5 nm or less. The absolute flatness of the surfaces may be measured through the use of the three-intersection method by near-infrared interferometry. This study represents an initial step toward the measurement of flattened silicon surfaces using a near-infrared interferometer.

## Abbreviations

CCD: Charge-coupled device; MRF: Magnetorheological finishing; PV: Peak-to-valley; RMS: Root-mean-square.

## Competing interests

The authors declare that they have no competing interests.

## Authors’ contributions

JU proposed a three-intersection method and analyzed the data. YH carried out the experiments of the three-intersection method using a near-infrared interferometer. NA fabricated the near-infrared interferometer. KK participated in the sample preparations. KA investigated the measurement accuracy. MM gave the final approval of the version to be published. All authors read and approved the final manuscript.
